# Spatial characteristics, similarity in business scope, and expansion network of listed companies in China’s food manufacturing industry

**DOI:** 10.1371/journal.pone.0351835

**Published:** 2026-06-30

**Authors:** Enkang Li, Yingyi Ma, Bo Hu, Wen Zhong, Ruoyan Zhang

**Affiliations:** School of Architectural Engineering, Jinling Institute of Technology, Nanjing, China; Beijing Normal University, CHINA

## Abstract

Using a multi-source dataset of 777 Listed Companies in the Food Manufacturing Industry (LCFMI), this study examines their spatial and relational organization in China. Key findings are: 1) The geographical distribution of LCFMIs closely aligns with China’s macro-economic landscape, with more developed regions hosting a greater concentration and larger scale of firms. 2) While similarity in business scope (SBS) is generally low, significant distance-decay effect is noted, particularly within “0 ~ xx km”; the resulting SBS network demonstrates small-world properties and distinct clustering patterns based on both geography and industrial sub-categories, highlighting the interplay between spatial and cognitive proximity. 3) The inter-city recruitment network, which reflects the spatial expansion of corporate influence, is predominantly shaped by transportation accessibility between cities. This research provides new empirical insights into the geographical organization of manufacturing and the formation of city networks, offering practical implications for regional industrial policy and infrastructure planning.

## Introduction

The food manufacturing industry is crucial for the national economy [1]. China has a large population and significant food demand. In recent times, its food manufacturing industry has grown rapidly, becoming a key sector in national economic construction and social development. In 2023, China’s total number of food manufacturing enterprises above a designated size reached 10,075, with total assets reaching RMB 2,163.9 billion [2]. Among them, the operating scale of listed companies in the food manufacturing industry (LCFMIs) is correspondingly larger because they can access financing support in the capital market, thereby exerting a greater impact on the daily lives of urban and rural residents in China. As a key sector in the manufacturing industry, the food manufacturing industry is comparable to other manufacturing sectors in its impact on regional economic patterns. For example, certain enterprises tend to expand from specific industries in economically developed cities, such as Beijing, to neighboring cities, thereby promoting the coordinated development of neighboring regions. Compared to other sectors such as pharmaceuticals, nano, and computers, the food manufacturing industry lacks strict requirements for the quality of the workforce and can absorb numerous people with low and middle levels of education, significantly increasing the incomes of urban and rural residents.

Despite the significance of LCFMIs, research on them remains limited, and several key questions remain unanswered: What are the distribution areas of these enterprises in China? What are the spatial layout’s characteristics of these enterprises? To what extent do the business scopes of various enterprises overlap? What forms of spatial expansion characteristics do enterprises exhibit during development? Addressing these questions is essential for comprehending the unique characteristics of China’s LCFMIs and informing macro-level policy planning. Therefore, aiming to bridge this gap in the literature, in this study a comprehensive research on this crucial topic is conducted.

## Theoretical framework

The core argument in this study centers on the Similarity of Business Scopes (SBS) among LCFMIs and the city network formed by cross-city recruitment of LCFMIs. Therefore, we developed a theoretical framework for understanding the spatial organization and cross-local connections of LCFMIs based on the theoretical traditions of economic geography and the analytical paradigm of city network theory. Specifically, we used the multi-dimensional proximity theory as the hub of the core argument to analyze the geographical spatial relationships among enterprises; at the same time, we applied the spatial interaction theory and gravity model to explore the city network formed based on the cross-city recruitment of LCFMIs and the influencing factors of network formation.

Our interest in the SBS among LCFMIs stems from long-standing concerns in economic geography about “agglomeration” and “distance.” Earlier studies mostly focused on geographical proximity and the factors influencing it, apart from the economic effects it would produce [3,4]. However, an increasing number of recent studies have begun to shift their attention to other dimensions of proximity, such as cognitive proximity, social proximity, and the driving mechanisms and policy effects behind this proximity. The SBS proposed in this study is an effective proxy variable for cognitive proximity, which can reflect the degree of proximity of enterprises in terms of knowledge base, technical capabilities, and market positioning. At the same time, a comprehensive analysis of this proximity and classic geographical proximity (i.e., geographical distance) has been conducted to explore (a) the relationship between SBS and geographical distance and (b) whether this relationship exhibits nonlinear characteristics as the geographical scale changes.

Enterprises aiming to avoid excessive competition from high SBS prefer to operate from different cities as their bases and not to cluster their operations in one city. However, this does not mean that they will easily abandon or ignore cities other than the headquarters location. They often establish branches and recruit employees in cities outside the headquarters location to expand their market influence as much as possible. This process strengthens the connections between the city where the headquarters is located and other cities. In other words, the recruitment behavior from the city where the headquarters is located to non-headquarters locations strengthens the spatial interaction between cities, making this an interesting and important aspect of urban network research over the years. In trying to explain this spatial interaction, the gravity model has been used successfully in many studies as a classic and suitable analytical tool.

**Fig 1:**
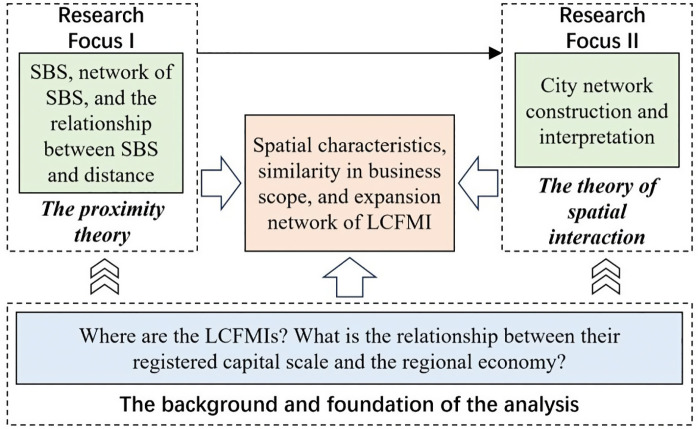
Framework of the research.

However, before conducting the analysis, we provide a comprehensive review of the overall situation of LCFMIs (such as the spatial pattern of LCFMIs and its relationship with the regional economy, etc.) to establish the background and foundation for the subsequent research. Therefore, following the logic of the above theoretical framework, we developed the following research framework (Fig 1). Our intention was to cover two core issues: (1) to discuss SBS and SBS networks, and the relationship between SBS and distance (theoretically supported by the proximity theory); and (2) to study the city network constructed based on the recruitment relationship and to analyze and explain the network using the gravity model (theoretically supported by the spatial interaction theory).

## Literature review

### Spatial layout of manufacturing enterprises and the theory of multi-dimensional proximity

The location selection of enterprises is a classic issue in the field of economic geography. For manufacturing enterprises, it necessitates a comprehensive consideration of the costs associated with spatial layout—including those related to labor and raw materials—as well as potential market returns. Generally, manufacturing enterprises tend to concentrate in regions with more developed transportation and higher capital intensity [5]. However, the priorities for location selection differ depending on firm size. Large enterprises value supervision, whereas Small and Medium-size Enterprises (SMEs) prioritize cost [6]. The behavioral characteristics of large enterprises are also obvious in multinational companies [7], as there are often certain risks in the selection and change of locations [8]. However, agglomeration is desirable. If parent and subsidiary companies are both located in a relatively developed metropolitan area, the production efficiency and economic benefits of the subsidiary company will be higher than those of subsidiaries far away from the parent company [9].

In China, state-owned and private enterprises in the manufacturing industry are related to each other in a spatial layout: upstream state-owned enterprises are in locations with higher enterprise concentration, and the entry degree of new private enterprises is significantly lower [10]. The spatial distribution of the manufacturing industry is affected by land policy [11], industrial structure, and the logistics level [12]. Several studies point to the core indicator, distance [13], which has been extensively discussed by many scholars in economic geography over the past hundred years. In addition, the agglomeration and dispersion of the manufacturing industry are closely related to producer services [14] and environmental issues such as carbon emissions in the region [15].

In fact, the location selection of manufacturing enterprises and the resulting spatial patterns are shaped by a combination of multidimensional factors, including distance and institutions. These investigations are closely related to the core concept of the theory of multi-dimensional proximity. This theory posits that the spatial layout of enterprises and the various relationships formed between them are influenced not only by geographical distance but also by other factors, including cognitive, organizational, and social dimensions. Moreover, the nature of this influence often varies depending on the type of enterprise. This leads to our point of interest: Does this pattern hold true for China’s food manufacturing industry?

### Network constructed based on “relationships”

Network analysis is an important analytical concept. City network areas are increasingly being studied in economics and geography. Inter-city connections can be categorized into networks formed by physical flows and those formed by relational constructions. Physical networks include the flow of vehicles, people, and goods between cities, whereas relational networks have no specific physical objects moving in space.

Relational networks include both cooperative and competitive relationships. Cooperative relationships, such as the city innovation network, can be constructed from patent [16–18] and thesis cooperation [19]. Competitive relationships, such as the city competition network [20], can be expressed by competition between cities for certain trade markets [21]. These relationships often have no apparent direction; however, similar to investment networks [22,23] and search engine-based relational networks [24], they are directional. Geographical distance further plays an important role in the formation of such relational city networks [25], with the distance decay effect widely observed and validated [26,27].

Descriptive analysis is fundamental in city network research. Social Network Analysis (SNA) is widely used for the specific calculation and description of network attributes [28–31], often supported by tools such as Gephi, UCINET, and Python. In econometric analysis or mechanism explanation, gravity models are commonly applied [32–35], while Quadratic Assignment Procedure (QAP) regression analysis is used to explore network formation mechanisms [36].

### Spatial interaction and the application of the gravity model

Spatial interaction is a pervasive phenomenon in economic geography. The interacting agents can be micro-geographic units such as firms, or macro-geographic units such as cities and nations.

Spatial heterogeneity serves as a fundamental driver of spatial interaction [37]. Such heterogeneity can induce the transfer of various elements—including population, goods, technology, and capital—from one location to another, analogous to water flowing from a higher to a lower elevation. The process of transfer itself constitutes the manifestation of interaction, implying a crucial logic: the interaction possesses directionality [38]. Certainly, factors driving interaction between cities or regions extend beyond mere spatial differences. Improved transport accessibility also plays a significant role in intensifying inter-regional interactions. For instance, the introduction of high-speed rail has been shown to facilitate the flow of production factors and expand the influence and radiating capacity of core cities [39]. Furthermore, interaction can also manifest as mutual dependence and coordination [40]. This spatial dependency is particularly evident between adjacent areas [41]. In such contexts, the application of spatial autocorrelation becomes a key analytical tool employed extensively in the literature to conduct effective empirical analysis [42].

However, merely describing an interaction is insufficient. We need to quantify how different factors influence this relationship. Undoubtedly, the gravity model stands as one of the most classic tools for analyzing such issues. Generally, many scholars adapt or modify the standard gravity model based on the specific characteristics of their research question to ensure that it more accurately reflects real-world contexts [43]. Selecting an appropriate estimation method is crucial for realizing the model’s full analytical value. A classical approach involves taking logarithms for linearization [44]. However, this method may not be useful in handling situations where the dependent variable contains zeros. Consequently, other important estimation strategies, including Poisson Pseudo-Maximum Likelihood (PPML) [45] and Negative Binomial Quasi-Generalised Pseudo-Maximum Likelihood (NB QGPML) [46], have become preferred alternatives in many studies.

### Summary

Therefore, the spatial layout of enterprises exhibits a propensity for agglomeration or dispersion. Different choices are related to the similarities and differences between firms. Distance is undoubtedly a crucial factor; together with other elements involved in the theory of multidimensional proximity, such as institutions and culture, it shapes the geographical distribution patterns of enterprises and indirectly influences the relationships between cities. This type of relationship can generally be regarded as spatial interaction, which can serve as a theoretical framework for expressing the expansion of urban influence. In-depth analysis and discussion of this phenomenon require the support of robust analytical tools such as network analysis and gravity model construction.

## Materials and methods

### Data

#### LCFMI.

The enterprise data for the collected 777 LCFMIs were derived from Tianyancha (https://www.tianyancha.com/). These include A-shares, Hong Kong stocks, US stocks, the new Third Board, and the new Fourth Board. All selected companies were still active as of December 19, 2024. The coordinate data of the, i.e.,s’ registered addresses were obtained from Baidu’s coordinate-picking system (https://api.map.baidu.com/lbsapi/getpoint/index.html).

Based on the raw enterprise data, we extracted and structured additional recruitment-related information based on the following criteria: First, the data records the recruitment announcements were published by enterprises in different cities; second, the recruitment announcements did not mention the number of specific personnel to be recruited for a certain position; and, third, enterprises had disclosed the relevant information on the Tianyancha platform. The data were analyzed as follows:

Suppose that $A_{\text{1}}$, an LCFMI registered in $C_{\text{1}}$ city, issues $n_{C_{\text{1}}A_{\text{1}}tC_{\text{2}}}$ recruitment announcement in $C_{\text{2}}$ city in year t, then the connection strength between $C_{\text{1}}$ city and $C_{\text{2}}$ city in year t can be defined as $\eta_{C_{\text{1}}tC_{\text{2}}}$. The formula is as follows:


$$\begin{array}{c} \eta_{C_{\text{1}}tC_{\text{2}}}=\sum_{i=1}n_{C_{\text{1}}A_{i}tC_{\text{2}}} \end{array}$$

Thus, a city network can be obtained based on the job posts. We can name such a network the LCFMI city network.

#### Economic data and inter-city transport accessibility data.

The urban gross domestic product (GDP) data were obtained from the China City Statistical Yearbook. Driving time data between cities, derived from the Baidu Map, were collected by the research team in May 17, 2021 (Monday).

### Method

#### SBS measurement.

The LCFMI data obtained from Tianyancha include the business scope of each enterprise. Following Manning et al. [47], we employed a comprehensive method based on Term Frequency-Inverse Document Frequency (TF-IDF) to calculate the similarity of business scopes among different LCFMI. The details are as follows:

1. TF-IDF Feature Extraction

TF-IDF feature extraction can quantify the importance of words in specific documents, balance the intra-document frequency and inter-document distribution of words, and generate numerical feature vectors for text. The relevant formulas are as follows:


$$\begin{array}{c} TF\left(d_{i},w_{j}\right)=\frac{count\left(d_{i},w_{j}\right)}{\sum_{k=1}^{K}count\left(d_{i},w_{k}\right)} \end{array}$$

$$\begin{array}{c} IDF\left(w_{j}\right)=log\left(\frac{M+1}{df\left(w_{j}\right)+1}\right)+1 \end{array}$$

$$\begin{array}{c} TF-IDF\left(d_{i},w_{j}\right)=TF\left(d_{i},w_{j}\right)\times IDF\left(w_{j}\right) \end{array}$$

where $d_{i}$ denotes the i-th document; $w_{j}$denotes the j-th valid word; $count\left(d_{i},w_{j}\right)$ is the number of occurrences of word $w_{j}$ in document $d_{i}$; K is the total number of valid words in document $d_{i}$; $\sum_{k=1}^{K}count\left(d_{i},w_{k}\right)$ is the total number of occurrences of all valid words in document $d_{i}$. M is the total number of documents in the document set; $df\left(w_{j}\right)$ is the document frequency of word $w_{j}$(the number of documents containing the word); log is the natural logarithm (to avoid excessive values); adding 1 to the numerator and denominator is a smoothing process to prevent the denominator from being 0 or the IDF value from being negative.

Based on the above information, we can construct the TF-IDF vector. The TF-IDF vector of document $d_{i}$ is:


$$\begin{array}{c} {𝐯}_{i}=[TF-IDF(d_{i},w_{\text{1}}),TF-IDF(d_{i},w_{\text{2}}),...,TF-IDF\left(d_{i},w_{V}\right)], \end{array}$$

where V is the total number of valid words in all documents (after stopword filtering and word pruning).

We calculate the cosine of the angle between the two TF-IDF vectors, quantify the semantic similarity between documents, and the result ranges from [0,1] (the larger the value, the higher the similarity). Let the TF-IDF vector of document $d_{i}$ be ${𝐯}_{i}=(v_{i1},v_{i2},...,v_{iV})$, and the TF-IDF vector of document $d_{j}$ be ${𝐯}_{j}=(v_{j1},v_{j2},...,v_{jV})$, where $v_{it}=TF-IDF\left(d_{i},w_{t}\right)$, $v_{jt}=TF-IDF\left(d_{j},w_{t}\right)(t=1,2,...,V)$. The cosine similarity between them is:


$$\begin{array}{c} Sim\left(d_{i},d_{j}\right)=cos\theta =\frac{{𝐯}_{i}\cdot {𝐯}_{j}}{{\|{𝐯}_{i}\|}_{\text{2}}\times {\|{𝐯}_{j}\|}_{\text{2}}}=\frac{\sum_{t=1}^{V}v_{it}\times v_{jt}}{\sqrt{\sum_{t=1}^{V}\overset{\text{2}}{\underset{it}{v}}}\times \sqrt{\sum_{t=1}^{V}\overset{\text{2}}{\underset{jt}{v}}}}, \end{array}$$

where ${𝐯}_{i}\cdot {𝐯}_{j}$ is the dot product of vectors ${𝐯}_{i}$ and ${𝐯}_{j}$; ${\|{𝐯}_{i}\|}_{\text{2}}$ and ${\|{𝐯}_{j}\|}_{\text{2}}$ are the L2 norms of vectors ${𝐯}_{i}$ and ${𝐯}_{j}$respectively; θ is the angle between the two vectors; V is the total number of valid words in all documents (consistent with the dimension of TF-IDF vectors). To facilitate the analysis, the value of cosine similarity is multiplied by 100, resulting in the SBS referred to in this study. The more detailed parameters involved in the calculation and their meanings can be found in S1 Appendix.

#### Social network analysis.

1. Degree of weight

The weighting degree refers to the sum of the weights of all the connections associated with a node. The formula used is as follows:


$$\begin{array}{c} K_{i}=\sum_{j}\varphi_{ij} \end{array}$$

where $K_{i}$ is the weighting degree of node iand $\varphi_{ij}$ is the weight of the connection between nodes i and j.

2. Community detection parameters

(1) Resolution parameter (γ)

Resolution is a core tuning parameter in modularity-based community detection that regulates the granularity of community division. It adjusts the algorithm’s preference for merging small communities or splitting large ones by modifying the penalty term in modularity calculation.

(2) Random seed

Random Seed (RS) is an initialization parameter for stochastic community detection algorithms. To ensure result robustness, multiple random seeds were tested to verify the stability of community structure.

3. Modularity (Q)

It is the primary metric for evaluating the quality of community division, quantifying the difference between the actual edge density within communities and the expected edge density in a random network with identical node degree distribution. The formula of Q:


$$\begin{array}{c} Q=\frac{\text{1}}{2m}\sum_{i,j=1}^{N}[A_{ij}-\gamma \frac{k_{i}k_{j}}{2m}]\delta \left(c_{i},c_{j}\right), \end{array}$$

where N is total number of nodes; m is total number of edges in the network (edges represent SBS between enterprise pairs); $A_{ij}$ is adjacency matrix element; γ is resolution parameter; $k_{i}$ is degree of node i (sum of SBS between enterprise i and all other enterprises); $\delta \left(c_{i},c_{j}\right)$ is indicator function (1 if enterprise i and j belong to the same community, 0 otherwise); $c_{i}$ is community label of enterprise i. Q>0 indicates that intra-community edge density is higher than that of a random network, confirming significant community structure.

4. Homogeneity index

Homogeneity Index is a metric measuring the consistency of node attributes (administrative regions/industry subcategories) within each community, reflecting the aggregation degree of enterprises by specific attributes (range: 0 = completely heterogeneous to 1 = completely homogeneous).

Formula of single community homogeneity (for the k−thcommunity) is:


$$\begin{array}{c} H_{k}=1-\frac{\sum_{t=1}^{M}\left(n_{k,t}lnn_{k,t}\right)}{n_{k}lnn_{k}} \end{array}$$

Formula of global homogeneity (weighted average across all communities) is:


$$\begin{array}{c} H_{\text{global}}=\sum_{k=1}^{C}\left(\frac{n_{k}}{N}\right)H_{k}, \end{array}$$

where C is the total number of detected communities; $n_{k}$ is number of enterprises in the k−th community; M is number of attribute categories (31 administrative regions/provinces; 7 industry subcategories); $n_{k,t}$ is number of enterprises in the k−th community belonging to the t−th attribute category.

5. Chi-Square Test

Chi-Square Test is a statistical test for validating the external validity of community structure, examining whether there is a significant association between detected community labels and enterprise attributes.

First, we need to formulate two mutually opposing hypotheses

H_0_: Community labels and target attributes are mutually independent (no association);
H_1_: Community labels and target attributes are significantly associated.

Formula is:


$$\begin{array}{c} \chi^{\text{2}}=\sum_{k=1}^{C}\sum_{t=1}^{M}\frac{(O_{k,t}-E_{k,t}{)}^{\text{2}}}{E_{k,t}} \end{array}$$

$$\begin{array}{c} DF=(C-1)(M-1), \end{array}$$

where $O_{k,t}$ is observed frequency (number of enterprises in the k−th community and t−th attribute category); $E_{k,t}$ is expected frequency under H_0_ ($E_{k,t}=\frac{n_{k}\cdot m_{t}}{N}$, where $m_{t}$ is the total number of enterprises in the t−th attribute category); The meanings of C, M, $n_{k}$, and N are the same as the corresponding content in the “Homogeneity Index” section. If p < 0.05, H_0_ will be rejected. This indicates a significant association between communities and attributes.

#### Gravity model.

The gravity model has been widely used in economic-geographical research. The basic form is as follows:


$$\begin{array}{c} F=\varphi \frac{{M_{\text{1}}}^{\alpha_{\text{1}}}{M_{\text{2}}}^{\alpha_{\text{2}}}}{D^{\beta }} \end{array}$$

Generally speaking, F represents the strength of the connection between cities or regions (such as the strength of the relationship and the flow of people); $M_{\text{1}}$ and $M_{\text{2}}$ are the “quality” of a city or region, which are generally expressed by GDP; D is mostly used to represent the spatial distance or traffic accessibility (e.g., the longer the driving time between the two places, the larger the D value, the lower the traffic accessibility level).

Compared with the strict assumptions of the Ordinary Least Squares (OLS) method regarding the distribution of error terms, the PPML estimation method has better applicability: it can not only naturally fit the non-negative integer type dependent variable, but also maintain estimation consistency when the dependent variable is a continuous non-negative value and excessive dispersion is present, and effectively handle the statistical interference caused by zero-value observations. Therefore, it is widely used in the empirical estimation of gravity models. In this study, we adopt this method, for which the formula is as follows:


$$\begin{array}{c} E\left[y_{ij}|{𝐗}_{ij}\right]=\text{exp}\left(\overset{\prime }{\underset{ij}{𝐗}}\beta \right)=\mu_{ij}, \end{array}$$

$y_{ij}$ represents the dependent variable (such as the strength of the connection between cities i and j); ${𝐗}_{ij}$ represents the explanatory variable vector (such as GDP, distance, etc.). β represents the vector of parameters to be estimated; and $\mu_{ij}$ represents the conditional expectation.

The log-likelihood function of PPML is:


$$\begin{array}{c} L\left(\beta \right)=\sum_{i,j}\left[y_{ij}ln\left(\mu_{ij})-\mu_{ij}-ln(y_{ij}!\right)\right] \end{array}$$

In this study, we used Python to write calculation programs for model diagnosis and regression estimation.

## Results

### Spatio-temporal characteristics of the distribution of LCFMIs

#### Overall pattern of LCFMIs in China.

LCFMIs were mainly distributed in the eastern and southern coastal areas of China, as well as in provincial capitals and their surrounding areas in the central and western parts of the country. Specifically, 34.49% of the LCFMIs were located in the eastern provinces (Beijing, Tianjin, Hebei, Liaoning, Shanghai, Jiangsu, Zhejiang, Fujian, Shandong, Guangdong, and Hainan), of which 49 had registered capital exceeding RMB 100 million—accounting for 42.61% of the national total. Moreover, 28.19% of the LCFMIs were located in central China (Shanxi, Henan, Anhui, Hubei, Hunan, and Jiangxi), of which 27 had registered capital exceeding RMB 100 million, comprising 23.48% of the national total.

Based on spatial distribution characteristics, the density and degree of agglomeration of the LCFMIs in different provinces varied. For example, the spatial distribution of the LCFMIs with high registered capital in Shandong was relatively not concentrated; however, that of the LCFMIs with high registered capital in Sichuan, Hunan, and other provinces was relatively concentrated. In Guangdong, the LCFMIs with high registered capital were mainly distributed in the Pearl River Delta region. These patterns reflect the spatial economic strategies and industrial priorities of the respective provinces. For example, Sichuan and Hunan showed prominent characteristics of a “strong provincial capital” strategy, where most of the investment and market resources are concentrated in provincial capitals, leaving other cities in the province relatively underdeveloped in terms of their economic, investment, and financing levels.

In addition, the number of LCFMIs in some provinces, such as Qinghai and Xizang, was small, and the amount of registered capital was low.

#### Distribution characteristics of the registered capital of LCFMIs by province.

**Fig 2:**
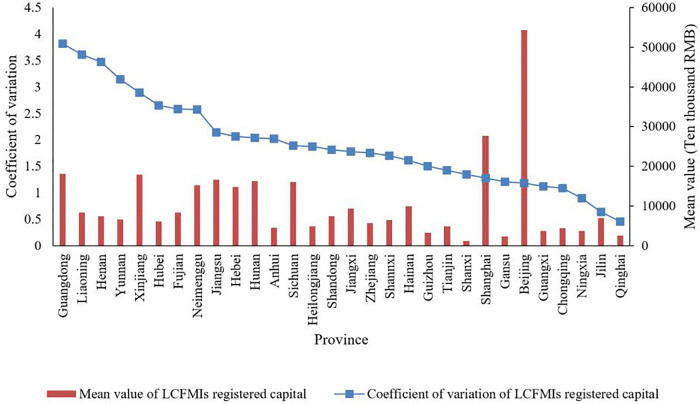
Coefficient of variation and mean value of LCFMI registered capital.

Fig 2 displays the variation in registered capital across provinces. The results showed a significant difference in the average registered capital of LCFMIs in different provinces, with that of Beijing and Shanghai being significantly higher than the others. Furthermore, the registered capital of LCFMIs within a province varied greatly in some cases and was relatively small in others. For example, the coefficient of variation for the registered capital of LCFMIs in Guangdong Province was 3.82, while that in Qinghai Province was 0.46.

**Fig 3:**
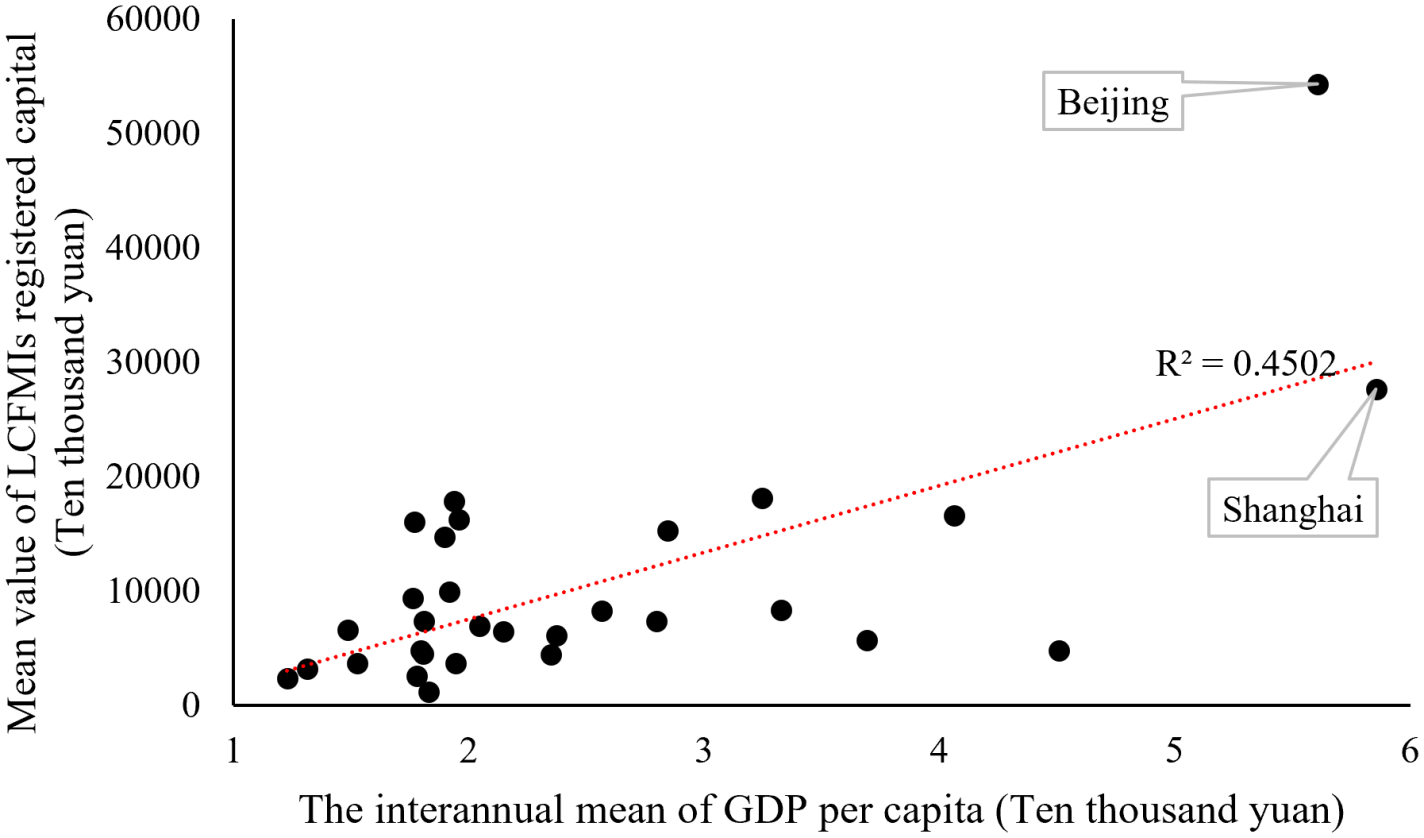
Relationship between provincial GDP per capita and LCFMI registered capital.

Overlaying the average registered capital of the LCFMIs with the average GDP per capita (Fig 3) of each province from 1981 to 2023, we found that the more developed the economy, the larger the scale of the LCFMI.

### SBS and network analysis

#### Overview of SBS.

**Fig 4:**
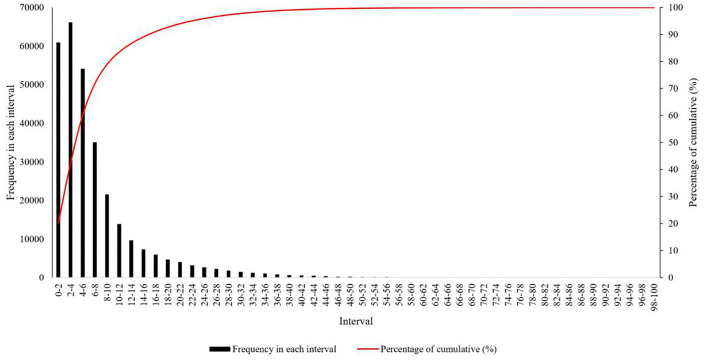
Segmentation and accumulation of SBS for different interval ranges.

Pairwise comparisons of company business scopes revealed a mean value of 7.23 and standard deviation of 8. Regarding distributional characteristics (Fig 4), 60.12% of pairs fell within the 0–6 SBS range, indicating that the vast majority of companies had large differences in the scope of their operations. In other words, the number of business segments in the LCFMIs was high.

#### Network of SBS.

We systematically examined the distinct community detection results of the SBS network under different algorithm selections and parameter configurations. As presented in Table 1, with the resolution parameter increasing from 1.0 to 1.1 and 1.2, the modularity value (Q) of the SBS network decreased from 0.13 to 0.09 and 0.07, while the number of communities increased from 3 to 6–9 and 9–11, respectively (across different algorithm and random seed combinations). This finding aligns with the general principle of network community detection, wherein higher resolution parameters tend to split cohesive communities into finer subgroups at the cost of reduced overall modularity.

**Table 1:** Properties of the SBS network under Different Parameter Settings.

Random seed	Algorithm	γ(Q, C)	γ(Q, C)	γ(Q, C)
50	Louvain	1 (0.13, 3)	1.1 (0.09, 7)	1.2 (0.07, 10)
	Leiden	1 (0.13, 3)	1.1 (0.09, 8)	1.2 (0.07, 10)
100	Louvain	1 (0.13, 3)	1.1 (0.09, 6)	1.2 (0.07, 10)
	Leiden	1 (0.13, 3)	1.1 (0.09, 6)	1.2 (0.07, 9)
150	Louvain	1 (0.13, 3)	1.1 (0.09, 9)	1.2 (0.07, 11)
	Leiden	1 (0.13, 3)	1.1 (0.09, 6)	1.2 (0.07, 10)
200	Louvain	1 (0.13, 3)	1.1 (0.09, 6)	1.2 (0.07, 10)
	Leiden	1 (0.13, 3)	1.1 (0.09, 8)	1.2 (0.07, 10)

The consistent trend of results generated by the Louvain and Leiden algorithms indicates robust community identification for this network, confirming that the detected community structure is an inherent attribute of the network rather than an artifact of algorithmic bias. Meanwhile, only minor fluctuations in community metrics were observed across different random seeds, further verifying the stability of the identified community structure.

To further explore the associations between the community division of the SBS network and firms’ administrative region affiliations as well as industry subcategories, this study calculated homogeneity indices to quantify the attribute consistency of nodes within each community and employed chi-square tests to assess the external validity of the community detection results. The resolution parameter of 1.0 was selected as the benchmark for in-depth analysis, as it represents the classical default setting for the Louvain algorithm and yielded a relatively high modularity value of 0.13. Under this resolution, both modularity values and community counts remained stable across different algorithms (Louvain and Leiden) and random seeds (50, 100, 150, 200). Additionally, the impacts of algorithm and random seed selections on community division were found to be negligible, supporting the prioritization of results derived from the Louvain algorithm with a random seed of 50 and resolution of 1.0 for external validity assessment.

The results revealed that the homogeneity index for administrative regions reached 0.54, indicating a moderate level of geographical agglomeration within the identified communities. In contrast, the homogeneity index for industry subcategories was relatively low at 0.27. Specifically, for administrative region distribution, firms located in Anhui, Fujian, and Guangdong provinces were significantly overrepresented in Community 0 compared to Communities 1 and 2, demonstrating a clear geographical clustering pattern. For industry subcategory distribution, all three communities were dominated by firms categorized under “Other food manufacturing” (208 firms in Community 0, 125 in Community 1, and 45 in Community 2). The high proportion of this category across all communities diluted the agglomeration effect of other specific industry subcategories. Notably, the “Other food manufacturing” category typically encompasses firms with broad business scopes, which are relatively difficult to define with precise industry boundaries.

### Relationship between SBS and distance

When discussing the distance decay effect and analogous issues, it is common practice to examine conditions across different distance intervals (bandwidths). In this study, we selected 40 bandwidths ranging from 5 km, 10 km, 15 km, …, to 190 km, 195 km, and 200 km (Distance here refers to the straight-line distance between companies.). For each bandwidth, we calculated the correlation coefficients between SBS and distance across distinct distance intervals, along with confidence intervals and significance levels under varying scenarios. To ensure the rigor of statistical inference, the Benjamini-Hochberg (FDR-BH) method was applied for multiple testing correction. Additionally, kernel smoothing analysis and correlogram analysis were conducted to corroborate the results derived from artificially defined bandwidths.

**Fig 5:**
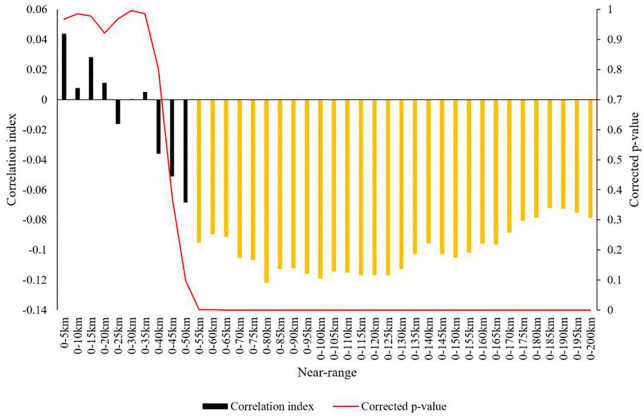
The correlation coefficients and corresponding p-values between SBS and distance for the “0 – xx km” group under different bandwidths.

We found that in most cases—regardless of whether the bandwidth was small or large—the corrected p-values corresponding to the correlation coefficients between SBS and distance within most distance groups generally exceeded 0.01 (Fig 5). However, when the bandwidth was 55 km or larger, the significance of the correlation coefficient between SBS and distance became highly pronounced in the first segment of the distance grouping (i.e., the range of “0~xx km”). This phenomenon indicates that the negative correlation between SBS and distance exhibits a distinct “near-range effect.” The small absolute value of the correlation coefficient in the near range suggests that while SBS and distance are significantly negatively correlated, the strength of this negative correlation is relatively weak.

**Fig 6:**
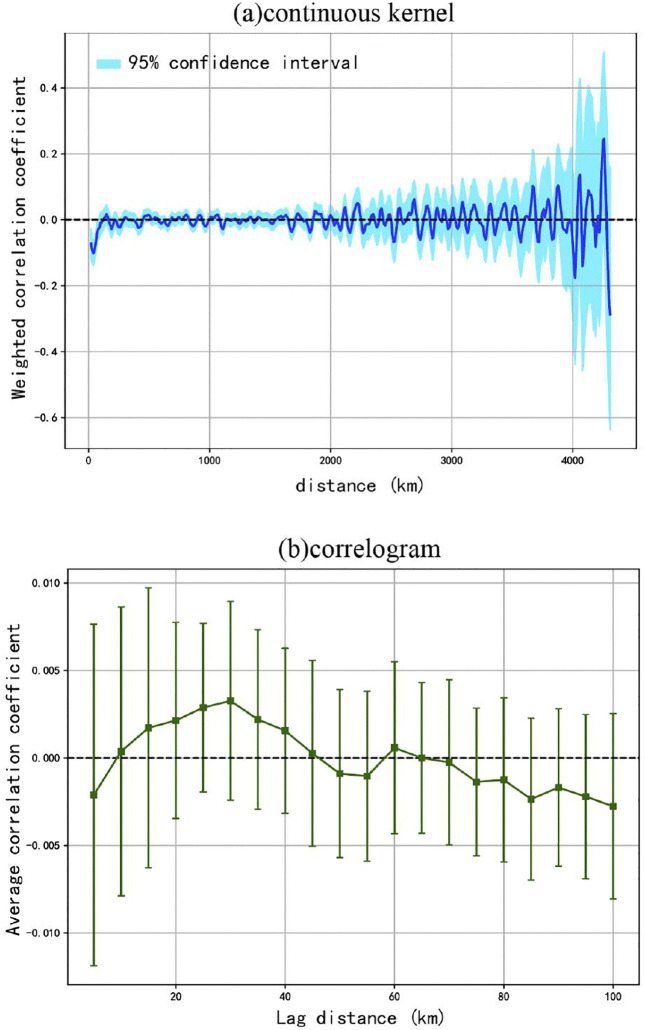
Continuous Kernel or Correlogram.

Our kernel smoothing analysis and correlogram analysis consistently support the above conclusions. As illustrated in Fig 6, within the 0–1000 km distance range, the blue solid line in the kernel smoothing plot shows minimal fluctuation, accompanied by narrow confidence intervals, indicating a weak but stable local correlation in the near-range region. In the correlogram analysis, the overall fluctuating downward trend demonstrates a degree of distance decay characteristics—weak yet consistently present.

### Formation and evolution of city networks in firm expansion

#### City network characteristics.

With the continuous development of their strengths, many LCFMIs are seeking to expand, and this movement is spatially manifested in the development of production and operational activities in cities other than the place of incorporation. This study used recruitment data to construct a city network reflecting such expansion.

**Fig 7:**
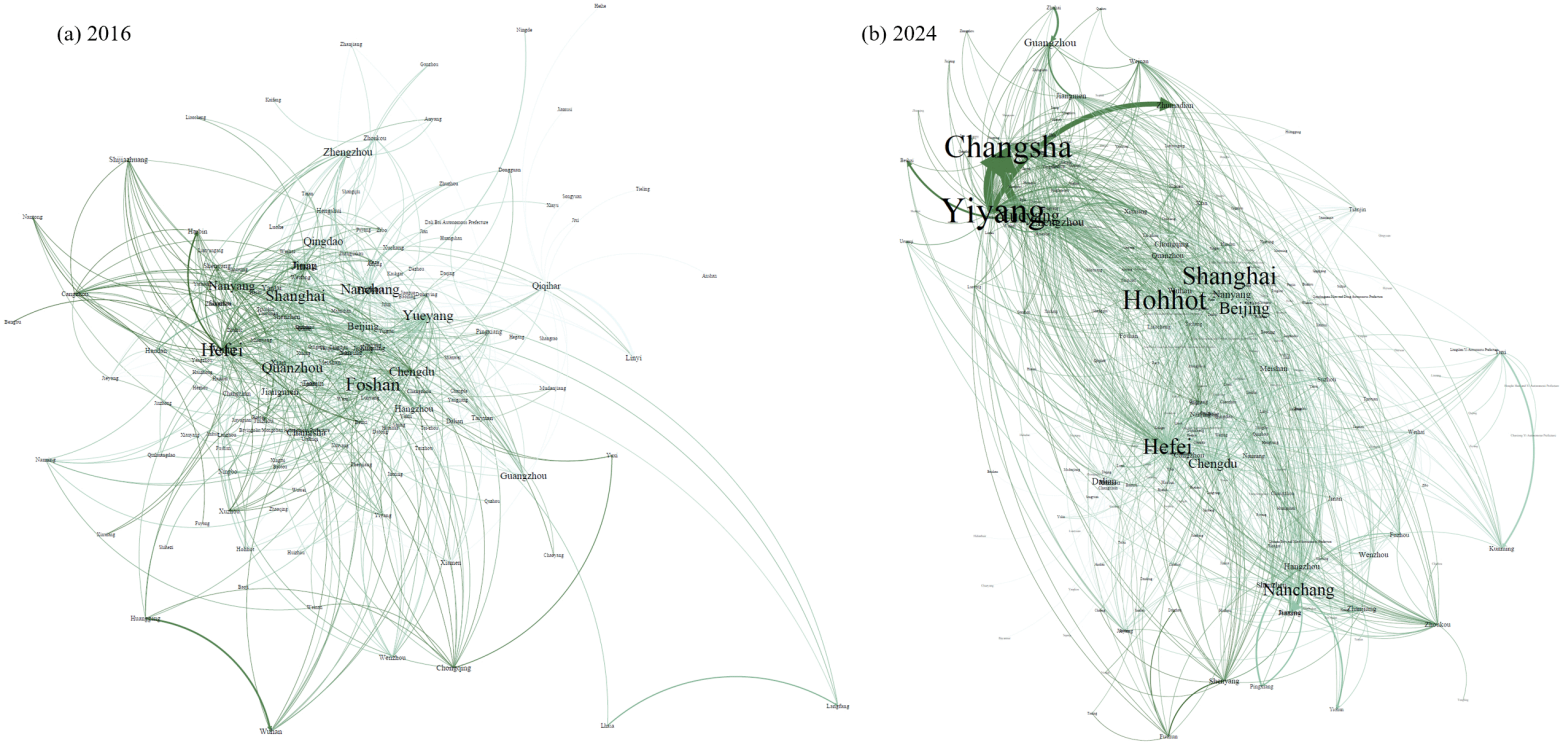
LCFMI city network: a) 2016 and b) 2024.

As shown in Fig 7, from 2016 to 2024, the LCFMI city network grew by 35.92% in terms of average weighting. The structure of the LCFMI city network in 2024 was more complex, with the emergence of sub-clusters such as “Changsha-Yiyang,” “Hohhot-Shanghai-Beijing,” and “Hefei-Chengdu.” These findings indicate that the development of LCFMIs further tightened inter-city relationships.

The node size represents the weighting degree and the node color indicates the module degree level; the darker and thicker the green line, the higher the weight of the edge.

#### Factors influencing the LCFMI city network.

This study refers to the research of scholars such as Larson [48], Kaya [49] and Larch [50]. When constructing the gravitational model to discuss which social and economic factors the LCFMI city network might be related to, the PPML method was adopted.

The connection strength of the LCFMI city network is set as y. Because the LCFMI city network is a directed network, the GDP of the starting city is *city1gdp*, and the GDP of the ending city is *city2gdp*. The driving time between the starting and ending cities represents the traffic accessibility between the two cities, which is set as *dr*. Whether the two cities belong to the same province (*sp*) and the distance (*d*) between the cities in a straight line will also have an impact on the connections between the cities; hence, they are also included in the model. When building the model, 1 is used to indicate that the two cities belong to the same province, while 0 is used to represent that they do not belong to the same province.

We employed four different methods to construct the gravitational model and then conducted diagnostics on them as well as compared the results. These four methods are: no fixed effect, fixed city1, fixed city2, and bidirectional fixed effect.

Before conducting the diagnosis of each model, we performed a multicollinearity test and found that the values of this item for each variable were all much less than 10 (*city1gdp*: 1.0153, *city2gdp*: 1.0117, *dr*: 1.1178, *sp*: 1.2309, *d*: 1.3422). In the specific diagnostic results (Table 2), Model 4 has no heteroskedasticity issue, and its McFadden_R² is the highest, reaching 0.4174, significantly exceeding the other three models. Meanwhile, the AIC and BIC values of Model 4 are also the lowest, while its log-likelihood is the highest among the four models. Therefore, based on the model diagnosis results, Model 4 is slightly better.

**Table 2:** Diagnostic results of the four models.

	Model 1 (No FE)	Model 2 (city1 Fixed)	Model 3 (city2 Fixed)	Model 4 (dyad FE)
**AIC (Akaike Information Criterion)**	68217.8241	54872.4783	60997.5744	45119.6710
**BIC (Bayesian Information Criterion)**	9559.0045	−2962.6799	4097.2067	−10957.0353
**Log-likelihood**	−34102.9121	−27304.2391	−30223.7872	−22158.8355
**McFadden_R² (McFadden Pseudo R-squared)**	0.1033	0.2821	0.2053	0.4174
**Pearson Chi-square**	218370.2568	109588.8464	86162.5766	45111.0401
**Degrees of Freedom**	5094	4968	4825	4699
**Overdispersion Coefficient**	42.8681	22.0589	17.8575	9.6001
**Overdispersion p-value**	0	0	0	0
**Durbin-Watson (Durbin-Watson Statistic)**	1.93418	2.0189	1.9228	2.0325
**Maximum Leverage**	0.0213	1	0.3129	1
**Mean Leverage**	0.0012	0.0259	0.0539	0.0786
**Maximum Standardized Residual**	234.9416	139.1309	105.3321	56.9002
**BP Test Chi-square**	15.5299	59.8016	167.8343	243.8237
**BP Test p-value**	0.0083	1	1	1
**Heteroskedasticity**	No	No	No	No
**Shapiro-Wilk Statistic**	0.2504	0.2517	0.3074	0.2828
**Residual Normality p-value**	0	0	0	0
**Same city1 Residual Autocorrelation**	N/A	N/A	N/A	N/A
**Same city2 Residual Autocorrelation**	N/A	N/A	N/A	N/A

However, from the perspective of the regression results, Model 4 still has its shortcomings. Although its Pseudo R^2^ is the highest, only one variable, *dr*, among the five variables is significant (p < 0.01), and the influence of *dr* on the dependent variable is negative (the coefficient is −0.0006, which is less than 0). In fact, all four models show significant results, and the coefficients are all negative. Therefore, for the LCFMI city network, a smaller value of *dr* is more conducive to strengthening the network connections. Another interesting issue highly worthy of discussion is the positive or negative nature of the coefficient of *d*. From Table 3, we observe that when p is less than 0.01, the coefficients of variable *d* in Model 1 and Model 3 are both positive, namely 0.0001 and 0.0002. This fact indicates that the relatively large straight-line distance between cities actually helps strengthen the internal connections within the LCFMI city network to a certain extent. The reason for this is that as information technology develops and transportation conditions improve, enterprises find it easier to establish personnel relationship networks across cities. Moreover, conducting recruitment and implementing production and business operations in a less developed city away from the headquarters can also help reduce costs and increase efficiency to a certain extent.

**Table 3:** Regression Results of the Four Models.

	Model 1 (No FE)	Model 2 (city1 Fixed)	Model 3 (city2 Fixed)	Model 4 (dyad FE)
**Intercept**	1.5195***(0.159)	0.8922	1.5471(6164.864)	0.8742
***city1gdp***	7.729 × 10^−6^**(3.45 × 10^−6^)	4.11 × 10^−5^(11.684)	9.832 × 10^−6^***(3.17 × 10^−6^)	4.938 × 10^−5^(10.511)
***city2gdp***	4.813 × 10^−5^***(3.72 × 10^−6^)	6.097 × 10^−5^***(3.22 × 10^−6^)	3.849 × 10^−5^	4.306 × 10^−5^
***dr***	−0.0002***(8.55 × 10^−5^)	−0.0005***(8.09 × 10^−5^)	−0.0003***(8.6 × 10^−5^)	−0.0006***(7.09 × 10^−5^)
***sp***	−0.3139**(0.127)	−0.1738(0.11)	−0.3237***(0.124)	−0.1269(0.104)
***d***	0.0001***(5.59 × 10^−5^)	−1.077 × 10^−5^(5.72 × 10^−5^)	0.0002***(5.49 × 10^−5^)	−2.251 × 10^−5^(5.55 × 10^−5^)
**Pseudo R** ^**2**^	0.7859	0.9851	0.9532	0.9980

## Discussion

Since China’s reform and opening up of its economy, the food manufacturing industry has advanced considerably. Based on their regional industry characteristics and inherent business strengths, various LCFMIs have established industry patterns with varying business scopes. This has resulted in significant market diversification, offering increasingly rich food consumption choices for urban and rural residents in China. Such diversification indicates significant segmentation within the industry and underscores the necessity of exploring the internal and external factors influencing a firm’s business scope in future research. This observation raises at least two critical questions. First, does SBS among LCFMIs reflect a pattern where latecomer firms learn from or imitate the forerunners? In other words, at the micro level, could it be that after observing Firm A’s substantial economic gains from certain food products, Firm B decides to locate closer to (to facilitate information sharing and infrastructure access) or farther away from (due to competition concerns) Firm A while simultaneously engaging in similar business activities? Second, do similar patterns exist among non-listed companies?

SBS exhibits an overall distance-decay pattern, which aligns with the spatial regularity of economic-geographic activities indicated by Rao et al. [51] and Han et al. [52] in their research. This indicates that the urban economic space has its own internal laws, which are often gradually formed after the comprehensive action and game of multiple factors, such as multiple market subjects and market economic systems and mechanisms. Regarding this point, a question worthy of further discussion emerges: Do similar phenomena exist across different manufacturing sectors? If so, how might the relationship between business scope similarity and distance in those sectors differ from the pattern observed in this study?

Because of their higher levels of informatization and financialization, listed companies, including those in the food manufacturing industry, have a greater potential for cross-city recruitment and expansion compared to small- and medium-sized non-listed enterprises. Such cross-city connections are governed by various factors, including inter-city traffic accessibility and urban economic scales. Therefore, it is essential to enhance and optimize traffic accessibility between cities. However, this aspect of research needs further development. More economic data should be gathered to enhance the model and improve its explanatory power in future research. Constructing urban networks based on inter-firm relationships is not limited to recruitment data. In fact, other critical linkages—such as supply chains between upstream and downstream enterprises, as well as inter-firm patent collaboration and technology transfer—can also serve as a foundational basis for capturing relationships between cities. This represents another promising avenue for future research.

As demand from Chinese urban and rural residents for high-quality food consumer goods increases, rational planning and guidance in the layout of food manufacturing enterprises in metropolitan areas, urban agglomerations, and other spaces become linked to whether the competition between production and operation among enterprises can be controlled within a reasonable range. From a policymaker’s perspective, various industrial policies must align with regional development to prevent congestion [53]. Specifically, the policy implications of this study are threefold. First, when planning food manufacturing clusters, policies should leverage the distance-decay pattern of business scope similarity. This means avoiding the introduction of firms with highly homogeneous businesses at very close spatial scales and instead fostering agglomerations based on industrial chain complementarity to optimize the competitive landscape. Second, given that transportation accessibility significantly influences the inter-city flow of factors, improving connectivity between core cities should be a priority for infrastructure investment. This enhances regional economic networks and facilitates knowledge spillovers. Third, considering the spatial hierarchy observed in the cognitive geography of firms, differentiated policy measures are necessary. Support for high-value-added activities should be focused around innovative cores, while specialized operations that utilize local resources can be encouraged in peripheral areas. This approach fosters regional synergy and complementary development.

This study has several limitations that should be acknowledged, which also point to directions for future work. First, our analysis focuses exclusively on listed companies. While listed firms are influential actors with significant impacts on regional economic landscapes—justifying their selection for this study—this focus necessarily excludes the vast population of small- and medium-sized, non-listed enterprises. Consequently, our findings regarding business scope patterns and inter-city networks may not be fully generalizable to the entire food manufacturing sector. Second, our dataset includes 777 LCFMIs that were active as of December 19, 2024. Although this date is 12 days before the end of the year, the proportion of firms likely to undergo major changes (such as de-listing) within this short window is minimal. Therefore, we consider the impact of this slight temporal discrepancy on our core conclusions to be negligible. Third, our modeling process involves certain simplifying assumptions. For instance, the selection of variables in the gravity model, though informed by theory, could be expanded. Future research could incorporate a wider array of factors influencing inter-city corporate linkages, such as detailed supply-chain data. Exploring these avenues would enhance the explanatory power and nuance of the model.

## Conclusion

This study, based on data including the location, registered capital, business scope, and recruitment announcements of 777 LCFMIs in mainland China, employs various methods such as text analysis, city network analysis, and the gravity model. It examines the distribution pattern of LCFMIs, the SBS network and its relationship with distance, the city network constructed from recruitment data, and the faxtors influencing this network. The main conclusions are as follows:

First, the distribution of LCFMIs aligns with the macroeconomic landscape of mainland China. More developed provinces host a larger number of companies with greater registered capital.

Second, the SBS values are generally low, reflecting the differentiated business strategies and models among LCFMIs. The SBS network constructed from pairwise similarities exhibits certain small-world characteristics, along with tendencies of spatial convergence and industrial subcategory convergence. This means that firms classified within the same community have a relatively high probability of being located in the same province, neighboring provinces, or belonging to the same industrial subcategory. A significant, albeit overall weak, negative correlation is observed between SBS and distance. This correlation is particularly notable within the “0 ~ xx km” segment.

Third, the city network constructed from corporate recruitment data reflects the spatial expansion of corporate influence and reach. The results from the gravity model analysis indicate that transportation accessibility plays a crucial role, suggesting that LCFMIs are sensitive to transport convenience when recruiting across cities.

## Supporting information

S1 File Supporting Information. (ZIP)

## S1 Appendix

The relevant parameters involved in calculating SBS, their meanings, and the calculation methods.

(1) Random Seed

Random seed can fix the initial state of the random number generator to ensure result reproducibility. Let the random number sequence be $R=\left\{r_{\text{1}},r_{\text{2}},...,r_{n}\right\}$, where $r_{k}\sim \;U\left(0,1\right)$(follows a uniform distribution over the interval [0,1]). By setting the seed s=42, the sequences generated in multiple runs are identical:


$$\begin{array}{c} \text{np.random.seed}\left(s\right)\Rightarrow \overset{\left(1\right)}{\underset{s}{R}}=\overset{\left(2\right)}{\underset{s}{R}}=...=\overset{\left(k\right)}{\underset{s}{R}} \end{array}$$

$\overset{\left(k\right)}{\underset{s}{R}}$ denotes the random number sequence generated with seed s in the k−th run; ⇒ means “is equivalent to”; $r_{k}$ is the k−th random number in the sequence (no conflict with symbols in other modules).

(2) Chinese Word Segmentation

We split continuous Chinese text into semantically independent word units, laying the foundation for feature extraction. Let the original text be$T_{i}=t_{i1}t_{i2}...t_{im}$($T_{i}$ is the original business scope text corresponding to document $d_{i}$, $t_{ik}$ is the k−th Chinese character in the text). The goal of word segmentation is to find the optimal word sequence $W_{i,raw}=\left\{w_{i1},w_{i2},...,w_{in}\right\}$ that satisfies


$$\begin{array}{c} \overset{n}{\underset{p=\text{1}}{\cup }}w_{ip}=T_{i},w_{ip}\cap w_{iq}=\emptyset (p\neq q)\text{arg}{\text{max}}_{W_{i,raw}}\overset{n}{\underset{p=\text{1}}{\Pi }}P\left(w_{ip}\right), \end{array}$$

where $W_{i,raw}$ is the original word set of document $d_{i}$ after segmentation; $w_{ip}$ is the p−th word in the set; $\overset{n}{\underset{p=1}{⋃}}w_{ip}$ denotes the union of all words (must cover the complete text $T_{i}$); $w_{ip}⋂w_{iq}=\emptyset$ means no overlap between any two words; $P\left(w_{ip}\right)$ is the occurrence probability of word $w_{ip}$ in the dictionary; ${argmax}_{W_{i,raw}}$ means finding the word sequence that maximizes the subsequent product.

(3) Stopword Filtering

Stopword filtering can remove words with no semantic contribution, retain valid features, and reduce subsequent computational complexity. Let the original word set of document $d_{i}$ after segmentation be $W_{i,raw}=\left\{w_{i1},w_{i2},...,w_{in}\right\}$, and the global stopword set be $S=S_{custom}⋃S_{len}$, where: $S_{custom}$ is custom industry stopword list (e.g., words with no differentiation such as “公司” [company], “服务” [service]); $S_{len}=\{w\left|len(w)<2\right\}$ is set of words with length less than 2 (len(w) denotes the character length of word w).

The valid word set of document $d_{i}$ afer filtering is:


$$\begin{array}{c} W_{i,valid}=W_{i,raw}-S=\left\{w_{ip}|w_{ip}\in W_{i,raw}\text{and}w_{ip}\notin S\right\} \end{array}$$

The global valid word set (union of valid words from all documents) is:


$$\begin{array}{c} W_{valid}=\overset{M}{\underset{i=1}{⋃}}W_{i,valid}=\left\{w_{\text{1}},w_{\text{2}},...,w_{V}\right\} \end{array}$$

$W_{i,valid}$ is the valid word set of document $d_{i}$; $W_{valid}$ is the total valid word set of all documents; V is the total number of valid words (consistent with the dimension of TF-IDF vectors); M is the total number of documents in the document set (consistent with M in IDF calculation).

(4) Document Frequency (DF)

Let the document set be $D=\left\{d_{\text{1}},d_{\text{2}},...,d_{M}\right\}$, and the global valid word set be $W_{valid}=\left\{w_{\text{1}},w_{\text{2}},...,w_{V}\right\}$. The document frequency of word $w_{j}$ is:


$$\begin{array}{c} df\left(w_{j}\right)=\sum_{i=1}^{M}I(w_{j}\in W_{i,valid}), \end{array}$$

where I(·) is the indicator function (if $w_{j}\in W_{i,valid}$ holds, i.e., word $w_{j}$ belongs to the valid word set of document $d_{i}$, then I=1, otherwise I=0); M is the total number of documents; V is the total number of valid words.

(5) N-gram Feature Extraction

We extract unigram (1-gram) and bigram (2-gram) features to capture word semantics and collocation relationships, enriching the dimension of text features.

Let the valid word sequence of document $d_{i}$ be $W_{i,valid}=\left\{w_{i1},w_{i2},...,w_{iK}\right\}$(K is the number of valid words in document $d_{i}$). Then:

1-gram feature set (unigrams): $G_{i,1}=\left\{w_{i1},w_{i2},...,w_{iK}\right\}$
2-gram feature set (bigrams): $G_{i,2}=\left\{w_{i1}w_{i2},w_{i2}w_{i3},...,w_{i(K-1)}w_{iK}\right\}$
Final feature set of document $d_{i}$: $G_{i}=G_{i,1}⋃G_{i,2}$

The global final feature set (union of features from all documents) is:


$$\begin{array}{c} G=\overset{M}{\underset{i=1}{⋃}}G_{i}=\left\{g_{\text{1}},g_{\text{2}},...,g_{V}\right\}, \end{array}$$

where $G_{i,1}$ and $G_{i,2}$ are the 1-gram and 2-gram feature sets of document $d_{i}$ respectively; $G_{i}$ is the final feature set of document $d_{i}$; G is the global final feature set; $g_{t}$ is the t−thfeature in the global feature set (corresponding to the word dimension in the TF-IDF vector, V is the total number of features, consistent with the previous total number of valid words); M is the total number of documents.

(6) L2 Normalization

We normalize the TF-IDF vector to eliminate the impact of document length differences on similarity calculation and improve result comparability. Let the TF-IDF vector of document $d_{i}$ be ${𝐯}_{i}=(v_{i1},v_{i2},...,v_{iV})$, where $v_{it}=TF-IDF\left(d_{i},g_{t}\right)$($g_{t}$ is the t−th feature in the global feature set G). The vector $\overset{\text{'}}{\underset{i}{𝐯}}=(\overset{\text{'}}{\underset{i1}{v}},\overset{\text{'}}{\underset{i2}{v}},...,\overset{\text{'}}{\underset{iV}{v}})$ after L2 normalization is:


$$\begin{array}{c} \overset{\text{'}}{\underset{i}{𝐯}}=\frac{{𝐯}_{i}}{{\|{𝐯}_{i}\|}_{\text{2}}}=(\frac{v_{i1}}{\sqrt{\sum_{t=1}^{V}\overset{\text{2}}{\underset{it}{v}}}},\frac{v_{i2}}{\sqrt{\sum_{t=1}^{V}\overset{\text{2}}{\underset{it}{v}}}},...,\frac{v_{iV}}{\sqrt{\sum_{t=1}^{V}\overset{\text{2}}{\underset{it}{v}}}}), \end{array}$$

where ${\|{𝐯}_{i}\|}_{\text{2}}$ is the L2 norm (Euclidean length) of vector ${𝐯}_{i}$; V is the total number of global features (consistent with the dimension of TF-IDF vectors and the total number of valid words); $\overset{\text{'}}{\underset{it}{v}}$ is the t−th element of the normalized vector.
