# Supplementary material for: Spatial characteristics, similarity in business scope, and expansion network of listed companies in China’s food manufacturing industry
Source: PLoS One. 2026 Jun 30;21(6):e0351835. doi: 10.1371/journal.pone.0351835 (PMC13318186; doi:10.1371/journal.pone.0351835)
Supplement: S1 File — (ZIP) [file pone.0351835.s001.zip › Access and download of Tianyancha data.doc]

1. Access the official website (<https://www.tianyancha.com/>) and log in (registration is required if you do not have an account).

2.

3. After setting the desired filters, you may proceed to click the search button. It is important to note that the screenshot below displays the query results from the page accessed on January 7, 2026 (Beijing Time). As you can see, compared to the data from December 19, 2024, the number of LCFMIs meeting the criteria has increased. If you are interested in the corporate data provided by Tianyancha, you may use this method to conduct your queries.
